# Population structure and genetic diversity in the nannandrous moss *Homalothecium lutescens*: does the dwarf male system facilitate gene flow?

**DOI:** 10.1186/s12862-015-0545-4

**Published:** 2015-12-03

**Authors:** Frida Rosengren, Bengt Hansson, Nils Cronberg

**Affiliations:** Department of Biology, Biodiversity, Lund University, Ecology Building, SE-223 62 Lund, Sweden; Department of Biology, Lund University, Molecular Ecology and Evolution Lab, Ecology Building, SE-223 62 Lund, Sweden

**Keywords:** Bryophytes, Genetic variation, Population structure, SNP, Long-distance dispersal

## Abstract

**Background:**

Nannandry is a sexual system where males (”dwarf males”) are much smaller than the conspecific females. Dwarf males occur in a wide range of unrelated organisms but the evolutionary advantages of this condition are poorly understood. The dwarf male sexual system results in differences in the mode of dispersal and establishment as well as the life span between males and females. Such differences must have profound effects on the population dynamics and genetic structures. We have studied four populations of the nannandrous moss *Homalothecium lutescens* in southern Sweden. We genotyped dwarf males and female shoots with the aim of describing the genetic diversity and structure of the populations.

**Results:**

Dwarf males were most related to their host shoot, then their colony (within 0.5 m^2^) and then the rest of the population, which suggests restricted spore dispersal. However, a few dwarf males in each population appeared to originate from other colonies and sometimes even other populations. Genetic diversity of dwarf males was generally high but showed no tendency to be consistently higher or lower than female genetic diversity within the four populations.

**Conclusions:**

Although most dwarf males have local origin, sporadic dispersal events occur. The ability of the dwarf males to establish in high numbers in mature colonies facilitates gene flow between populations as well as increases the potential to accumulate genetic diversity within populations.

## Background

Nannandry, i.e. the presence of dwarf males, is a breeding system that has evolved in many different organism groups and environments [1, 2]. For such a sexual system to evolve, small males need to have higher fitness relative to larger males. Such advantages could arise if fertilization success is increased when dwarf males are present as epiphytes on females in close vicinity to the female reproductive structures, as is the case in mosses [3, 4] and barnacles [5]. Alternatively, sexual maturation may be speeded up at the expense of growth and development, as is the case in certain annelids [6] and velvet worms [7–9]. To understand the evolution of the dwarf male sexual system, we need to understand how the dwarf males affect the genetic variation and structure within populations, both spatially and over time, through processes like gene flow, inbreeding and outcrossing.

Among photosynthesizing organisms, dwarf males only exist in aquatic green algae (order Oedogoniales) [10] and mosses. More and more moss species are discovered to have dwarf males and it is estimated that dwarf males occur in 10–20 % of all moss species (and in at least a fourth of all unisexual pleurocarpous mosses) [11]. Despite the fact that dwarf males in several moss species represent the majority of male individuals in the population, they have to date not been taken into account when describing genetic structure and diversity in moss populations. As a matter of fact, genetic studies of epiphytic dwarf males (in any organism) are almost lacking (but see [12, 13]).

Mosses differ from vascular plants in that the dominant life stage (i.e. the shoot) is haploid, the only stage that is diploid is the sporophyte. Roughly half of all moss species are unisexual, that is the male and female sexual organs are borne on different individuals [14]. In unisexual mosses, dwarf males originate from male spores that land and germinate on the female shoots, where their final size is restricted to a few millimetres. Large males (presumably originating from male spores that have germinated on the ground) can be found to various extents in nannandrous mosses. In the focal species of this study, large males are extremely rare and are therefore not taken into consideration [4, 15, 16]. Having the males growing as tiny epiphytes on the females facilitates fertilization, which in mosses is mediated by swimming spermatozoids [17]. Dwarf males in mosses differ from large shoots in several aspects: 1) Their life span is most likely restricted to 1–2 years [3, 4, 11, 18–20], which means they must be recruited every year, whereas large shoots are often perennial. 2) They may readily establish within mature moss colonies since the competition for space is minimal and substrate is always present, whereas spores settling on the ground require some sort of disturbance. 3) They may cluster in large numbers in a comparably small space, while large shoots are expanding by vegetative growth over larger areas. 4) They primarily disperse by spores, which contrasts with the situation in female shoots that may primarily disperse locally by detached vegetative fragments. For these reasons, it is necessary to take both dwarf males and the “normal-sized” parts of the population into account when analysing genetic diversity and genetic population structure.

Moss spores are primarily wind-dispersed and have a leptokurtic dispersal distribution [21–23]. Consequently, spores that develop into dwarf males potentially have a long-range origin, but in colonies with abundant sexual reproduction the majority of dwarf males on a shoot likely originate from spores produced in the same colony or even on the female host shoot [11, 18, 24, 25]. Thus, the dwarf male sexual system potentially both facilitates long distance gene flow and increases inbreeding. Observations of dwarf male distribution and population genetic structure present a unique opportunity to study the efficiency of spore dispersal, which may be difficult to access by other means.

The first and only study of moss dwarf male genotype diversity found some variation in the ITS regions among dwarf males from a single female host [12]. They also found indications of one dwarf male originating from a non-host female, suggesting that a single female may host a variety of dwarf males. However, the interpretation of their results was limited by small sample size and genetic markers with low resolution. With improved DNA extraction and genotyping technology, it is now possible to genotype dwarf males in large numbers and at many markers. For the first time, we present a study of moss genetic population structure and genetic diversity that includes both dwarf males and normal-sized female shoots. We have studied four populations of the nannandrous moss *Homalothecium lutescens*, all with a high production of spores and abundant presence of dwarf males, at a set of 68 single-nucleotide polymorphism (SNP) markers. In our study we aim to answer the following questions: 1) What is the genetic structure of dwarf males and females, within and between populations? 2) How are the dwarf males related to their female host shoot? 3) Are the levels of genetic diversity similar in female shoots and dwarf males? By answering these questions we hope to get a better understanding of the potential evolutionary advantages of the dwarf male sexual system.

## Methods

### Study species

*Homalothecium lutescens* is a perennial pleurocarp that proliferates largely by clonal growth. It can be found mainly on the ground in calcareous, open habitats. Large males in *H. lutescens* are rare; instead the majority of males are situated as tiny epiphytic dwarf males on the large female shoots [26]. The dwarf males are situated behind the leaves close to the stem (either on the main stem or side branches, usually not on branch tips), on the mid section of the shoot. Although we observed dwarf males on large shoots all year round, the prevailing opinion is that dwarf males in general are not able to live more than one or maximally two years on the large shoots [3, 4, 11, 18–20].

### Sites

**Fig. 1:**
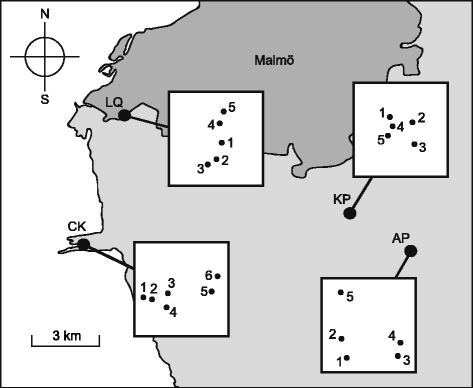
Locations of four sampled populations of the moss *Homalothecium lutescens*. Four populations of the moss *Homalothecium lutescens* in southern Sweden close to the city of Malmö (55° 34.8’ N 13° 0’ E): Arrie ponds (AP), Käglinge ponds (KP), Limhamn Quarry (LQ) and Cape Klagshamn cape (CK). The dots within the squares represent the colonies sampled within each population (drawn to the same scale, square height/width 230 m). Colony numbers are equivalent to the ones used in the structure plot (Fig. 4)

Sampling was conducted in four different populations in southern Sweden where dwarf males and sporophytes had previously been found in abundance: 1) Arrie ponds (AP, 55°31’15.3”N 13°6’5.7”E), 2) Käglinge ponds (KP, 55°31’57.2”N 13°3’57.6”E), 3) Limhamn quarry (LQ, 55°34’5.7”N 12°55’29.0”E) and 4) Cape Klagshamn (CK, 55°31’21.0”N 12°54’2.8”E). AP is a partly forested, hilly, recreational area that has previously been used as a gravel pit. *Homalothecium lutescens* is abundant in large areas of AP, mainly where the ground is more open. KP is a location similar to AP in use, vegetation and topography. In KP, *H. lutescens* occurs mainly in a small (0.3 ha), hilly area next to some ponds. LQ consists of the bottom of a large (188 ha and 65 m deep) former opencast pit. In LQ, *H. lutescens* occurs most abundantly in a small (4 ha) area that is partly covered with trees and shrubs. CK is an artificial peninsula created with residues from chalk and limestone industry. The area is partly forested; *H. lutescens* mainly occurs in areas where trees and shrubs have been cleared. All four populations are located within an area of approximately 60 km^2^ (Fig. 1), which mainly consists of arable land and suburbs of the city of Malmö.

### Sampling

**Fig. 2:**
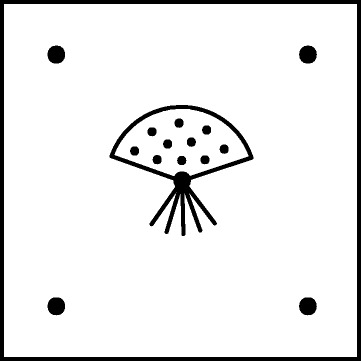
Sampling within colonies of the moss *Homalothecium lutescens*. Large black dots represents female shoots (*five in each colony*). From the centre female shoot, an average of five sporophytes (*lines radiating from dot*) and ten dwarf males (*small black dots*) were sampled. The distance between the centre female shoot and the other female shoots were 50 cm

Five colonies (six in population CK) were selected in each of the four populations and sampled in November 2012. The colonies were chosen for having as many sporophytes as possible (up to 20 per shoot) and being at least 20 metres apart from each other. From each colony, five 5 × 5 cm moss samples were taken, one sample in the centre and four samples 50 cm from the centre in four separate directions (similar to the dot pattern of number five on a dice, Fig. 2). All samples should contain at least one sporophyte. In the lab, the central sample of each colony was examined and the first shoot found with at least four sporophytes and at least ten dwarf males was selected as “centre shoot.” The shoots from population CK had relatively few dwarf males, thus, six colonies were chosen from this population for further analyses to increase the chances of getting DNA from enough dwarf males in this population. Finally, one random shoot from each of the four remaining samples in each colony having at least one sporophyte was selected. In total, 105 female shoots were selected for further analyses, 25 from each population (except for KP where 30 shoots were selected). The sampling area (polygon) in the four populations differed, in AP it was 13 891 m^2^, in KP it was 2 816 m^2^, in LQ it was 1 546 m^2^ and in CK it was 4 749 m^2^. The mean distance to the closest sampled colony was 56 m in AP, 35 m in KP, 30 m in LQ and 32 m in CK.

### DNA extraction

Shoots were kept in open top plastic test tubes in a growth chamber and were regularly sprayed with distilled water to prevent desiccation. DNA extraction was performed with Qiagen DNeasy Plant MiniKit. Between four and six sporophytes from each centre female shoot were selected for DNA extraction. In addition, between ten and twenty dwarf males from each centre female shoot were placed on agarose plates made with with a weak nutrient solution [27]. Because of algal and fungal contamination, dwarf males were transferred to new agarose plates approximately once a month. After about four months, dwarf males had grown to between 3 mm and 3 cm long (the longer ones were somewhat etiolated) and DNA extraction was performed. In total, DNA extraction was performed for 108 diploid sporophytes, 239 dwarf male haploid gametophytes and 105 female haploid gametophytes. The data from the sporophytes are analysed and presented in a parallel paper [28].

### SNP genotyping

68 SNPs, extracted from a 454-sequenced transcriptome of *H. lutescens*, were used for genotyping (thoroughly described in another paper) [28]. SNP-typing was performed by SciLifeLab Uppsala with the Illumina GoldenGate assay [29].

### Population structure analyses

Principal coordinate analysis (PCoA) of female shoots and dwarf males in all four populations was performed using the Excel plugin GenAlEx (version 6.5) [30, 31]. The analysis were based on the pairwise genetic distance (i.e. number of loci with differing alleles) matrix, with data standardization (i.e. division by the square root of n-1). Resulting graphs were edited in Adobe Illustrator CS6.

The software STRUCTURE (version 2.3.4) was used to detect within and between population structure [32]. Data was entered in haploid form. We used an admixture, correlated allele frequencies model and a priori information about population (AP, KP, LQ and CK) to determine the most likely number of population clusters K. We set K from 1 to 24 using a burn-in of 20 000 and 50 000 replications. Analyses were repeated 15 times for each run of a certain K. The average and standard deviation (SD) of the likelihood of the model of each K was used to calculate ∆K [33] in Structure Harvester [34]. The models with the highest and second highest ∆K were selected as the models with the most support. CLUMPP (version 1.1.2) was used to align the 15 repetitions for each selected K, using G’, Greedy algorithm for K = 4 and LargeKGreedy algorithm for K = 15 (other options as default) [35]. Results from CLUMPP were visualized by the Distruct software (version 1.1) [36] and edited in Adobe Illustrator CS6.

### Relatedness between dwarf males and female shoots

The differences in genetic distance (i.e. the proportion of differing alleles) between individual dwarf males and female shoots were tested in two linear mixed effects models: 1) Comparing genetic distance between dwarf males and female host shoot (GD_HOST) with the genetic distance between dwarf males and the remaining four sampled female shoots in the same colony (GD_COL). 2) Comparing GD_COL with the mean genetic distance between dwarf males and the rest of the sampled female shoots in the population (GD_POP). Data (throughout the paper) are given as mean ± SD (standard deviation).

Linear mixed effects models were constructed with the lmer function (lme4 package) [37] in R version 3.1.1 [38] with genetic distance as a response variable. Genetic distance category (two levels, GD_HOST and GD_COL or GD_COL and GD_POP) was treated as a fixed effect. Population, colony (nested within population) and individual were treated as random effects. Response variable (genetic distance) in model 1 was square root transformed to meet the model assumptions (normal distributed residuals and homogeneity of variances). Model assumptions were checked with the function mcp.fnc (R package LMERConvenienceFunctions) [39], which plots density of model residuals, QQ-plot and fitted values versus the standardized residuals. P-values were obtained with the R package lmerTest [40].

### Between and within population genetic differentiation

Genetic differentiation between populations was quantified with *Φ*_PT_ (calculated via AMOVA, an analogue of *F*_ST_ for haploid data) in GenAlEx version 6.5 [30, 31], for dwarf males and shoots separately. As individual samples within colonies are not independent and dwarf males and female shoots are not sampled in a similar manner, dwarf males and females were divided into five subsamples each per population. Each subsample of dwarf males or female shoots consisted of a unique dataset of one individual per colony (i.e. *N* = 5 in populations LQ, KP and AP while *N* = 6 in population CK). *Φ*_PT_ was calculated for all ten subsamples (five subsamples of dwarf males and five subsamples of females), and the difference between *Φ*_PT_ in dwarf males and females was tested in a t-test in R version 3.1.1.

Genetic differentiation between colonies within populations was quantified with *Φ*_PT_ for dwarf males and female shoots (all individuals included). Note, however, that as the sampling areas within colonies are different for female shoots and dwarf males (0.5 m^2^ versus one host shoot), the levels of within population differentiation are mainly descriptive and cannot be directly compared between dwarf males and females.

### Correlation between genetic and geographical distances

Pairwise kinship coefficients *F*_*ij*_ [41] were calculated for all pairs of individual dwarf males and all pairs of individual female shoots, with the software SPAGeDI v. 1.5 [42]. Kinship (*F*_*ij*_) is expected to decrease as the geographic distance between individuals increases. A possible association between the matrix of kinship coefficients and the matrix of geographic distances was calculated with a Mantel test (999 permutations) [43] in GenAlEx version 6.5 [30, 31]. Tests were performed for dwarf males and female shoots separately, both on an overall level (individuals from all populations included) and within each population.

### Clonal and genetic diversity in dwarf males and female shoots

Genetic diversity in dwarf males and female shoots on population level were estimated in two variables: Nei’s unbiased diversity (uh = (n/(n-1)) × (1- Σ*ρ*_*i*_^2^), where *ρ* is the frequency of the *i*th allele) [44] and proportion polymorphic loci (%P), calculated with GenAlEx version 6.5 [30, 31]. Due to the fact that dwarf males and shoots within colonies are not independent samples, genetic diversity estimates on population level were based on a mean of 1000 permutations with one random individual from each colony sample (dwarf male or shoot). The randomized datasets were constructed using R version 3.1.1 [38].

The haploid SNP genotypes (the combination of alleles in a haploid gametophyte) will hereafter be referred to as haplotypes. The number of distinct haplotypes per sampled shoot (Hap), uh and %P were calculated for dwarf males and female shoots separately on colony level, all individuals included. For Hap, individuals that share haplotype over all loci except for where there are missing data were treated as the same haplotype. To examine whether the diversity of the dwarf males on a female shoot is dependent on the diversity of the females within the colony, the relationship between dwarf male diversity (dependent variable) and female shoot diversity (fixed factor) within each of the three diversity variables (Hap, uh and %P) was tested in linear mixed effects models with the lmer function in R version 3.1.1 [38]. Population was treated as a random variable.

## Results

### SNP-genotyping

All samples except 30 dwarf males could be SNP-genotyped. In total, 105 female shoots and 209 dwarf males (mean 10.0 ± 2.1 dwarf males per female host shoot, ranging between five and twelve) were genotyped. Total proportion of missing data among the genotyped samples (failed genotyping) was 1.1 %, more or less evenly distributed among individuals and loci. No individual had more than 10 % missing data and no loci had more than 17 % missing data. The number of polymorphic loci differed between populations: 66 in AP, 55 in KP, 48 in LQ and 56 in CK. The samples from population AP contained four alleles that were unique for this population; none of the other populations had any unique alleles.

### Between- and within-population structure

**Fig. 3:**
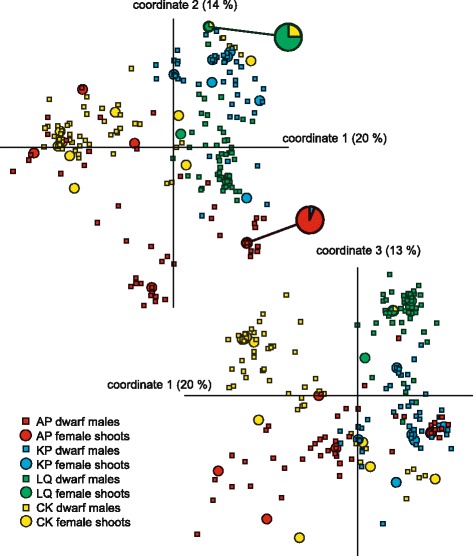
PcoA of four populations of the moss *Homalothecium lutescens*. Results from a principal coordinate analysis (upper graph: axes 1 and 2; lower graph: axes 1 and 3) based on a genetic distance matrix of female shoots and dwarf males (314 individuals) in four populations (AP, KP, LQ and CK) of the moss *Homalothecium lutescens* in southern Sweden. Two female haplotypes (circles with thicker outline, enlarged in the upper graph) are present in two different populations (AP/KP and CK/LQ), and are colour coded according to the proportion from each population (in both occasions, the smaller proportion represents a single shoot)

**Fig. 4:**
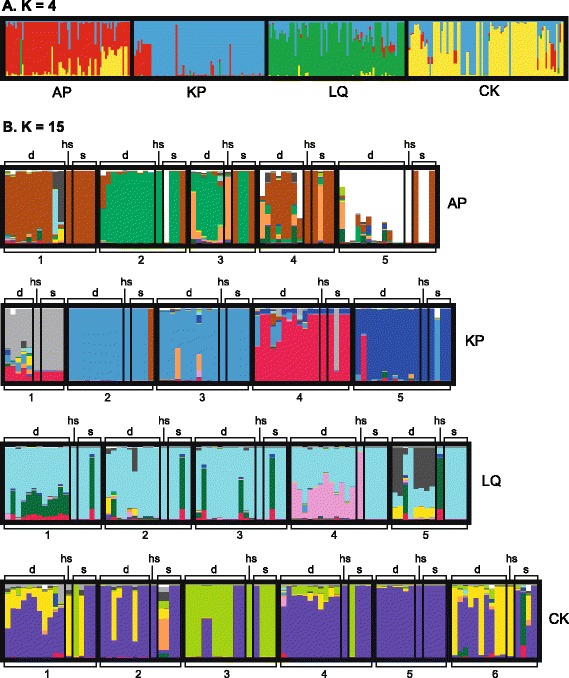
STRUCTURE plots of four populations of the moss *Homalothecium lutescens*. The population genetic structure in the moss *Homalothecium lutescens* from analyses with the programs STRUCTURE and CLUMPP for (A) K = 4 and (B) K = 15 (*divided by population*). Each plot was based on 68 SNPs from 314 individuals in four populations (AP, KP, LQ and CK). The plots are read from left to right and are sorted by population and colony (5 colonies in AP, KP and LQ, 6 colonies in CK), with bars representing individual admixture proportions to different genetic clusters (represented by different colours) (d = dwarf males, hs = host shoot, s = female shoots)

Each of the four populations (AP, KP, LQ and CK) formed different clusters in the PCoA, with varying degree of overlap (Fig. 3). The first three axes explained 47 % of cumulative variation (1: 20, 2: 14 % and 3: 13 %). STRUCTURE runs with subsequent analysis in Structure Harvester revealed K = 4 to be most likely (∆K = 28.03), generally corresponding to the four populations (Fig. 4a). There was no difference in between-population differentiation between dwarf males (mean *Φ*_PT_ = 0.286 ± 0.088) and female shoots (mean *Φ*_PT_ = 0.374 ± 0.161) (*t*_[6.19]_ = −1.07, *p* = 0.325).

STRUCTURE runs revealed K = 15 to be the second most likely number of genetic clusters (∆K = 10.92), corresponding to varying degrees of differentiation between colonies within the four populations (Fig. 4b). Genetic differentiation between colonies (*Φ*_PT_) was stronger in dwarf males than in female shoots in all populations except KP (where differentiation between female shoots was exceptionally high) (Table 1). A stronger differentiation between dwarf males in different colonies was more or less expected since the dwarf males within colonies are sampled from smaller area (a single host shoot) compared with the female shoots (0.5 m^2^). Population LQ stands out with no genetic differentiation between the female shoots in the different colonies (Table 1).

**Table 1:** Genetic differentiation between colonies within four populations of the moss *Homalothecium lutescens* in Sweden

Population	Category	*N*	*Φ*_PT_	*P*
AP	Dwarf males	47	0.504	0.001
	Female shoots	25	0.230	0.019
KP	Dwarf males	50	0.644	0.001
	Female shoots	25	0.707	0.001
LQ	Dwarf males	53	0.248	0.001
	Female shoots	25	−0.167	1.000
CK	Dwarf males	59	0.286	0.001
	Female shoots	30	0.226	0.014

Values (*Φ*_PT_, via AMOVA) are calculated for dwarf males and female shoots separately. Dwarf males within colonies are sampled from a single female shoot, while female shoots are sampled from an area of 0.5 m^2^. *N* = total sample size. Number of colonies is five in each population except in CK where it is six. *P* = significance level determined with permutation test (999 permutations); note that all *Φ*_PT_ -values are significant (i.e. *P* < 0.05) except for female shoots in pop LQ

The kinship coefficient *F*_*ij*_ decreased with geographic distance in both dwarf males and female shoots on an overall level (individuals from all populations included) (Table 2). Within populations, a negative association between kinship and geographic distance in the female shoots could be detected in one out of four populations (pop CK, which also had the strongest differentiation between colonies, *Φ*_PT_). In the dwarf males, a similar negative association could be detected in three out of four populations (Table 2).

**Table 2:** Result of Mantel tests comparing individual kinship coefficients *F*_*ij*_ and geographical distances in four populations of the moss *Homalothecium lutescens*

	pop	*N*	*r*	*P* (999 permutations)
Dwarf males	AP	47	−0.271	<0.001
	KP	50	−0.330	<0.001
	LQ	53	−0.042	<0.001
	CK	59	0.069	0.864
	ALL	209	−0.372	<0.001
Female shoots	AP	25	0.120	0.945
	KP	25	−0.131	<0.001
	LQ	25	0.274	0.219
	CK	30	−0.202	0.175
	ALL	105	−0.478	<0.001

### Relatedness between dwarf males and their host shoot

The genetic distance between the dwarf males and their host shoot (GD_HOST) was lower compared to the genetic distance between the dwarf males and the female shoots in the rest of the colony (GD_COL) (*F*_[1,208]_ = 64.57, *p* < 0.001, estimate = −0.70). The genetic distance between the dwarf males and the female shoots within the same colony excluding host shoot (GD_COL) was lower compared with the genetic distance between the dwarf males and the female shoots in the rest of the population (GD_POP) (*F*_[1,396]_ = 197.9, *p* < 0.001, estimate = −5.80). However, there are individual exceptions to the pattern of dwarf males being most similar to their host shoot (or at least their own colony) (Fig. 4a and b). In population AP, at least one dwarf male (possibly two) in colony one, are more similar to population LQ. In population KP, two dwarf males in colony three appear to show some similarity with population AP. Within populations, there were several examples of dwarf males that appear to originate from a female shoot other than the host (either from their own colony or another colony within the population) (Fig. 4b).

### Clonal and genetic diversity

A total of 22 different female haplotypes were found (from 105 samples). Seven haplotypes were found only once, three haplotypes were found twice, nine haplotypes were found between four and six times, three haplotypes were found 14, 15 and 20 times, respectively. A majority of the haplotypes (64 %) were confined to a single colony, but on numerous occasions identical female haplotypes were found in more than one colony within a population. Two of the haplotypes were found in two different populations: one in populations KP (one shoot) and AP (14 shoots) and one in populations CK (one shoot) and LQ (four shoots). Within colonies (0.5 m^2^), the number of distinct female haplotypes ranged between one (all five samples identical) and five (all unique), with a mean of 2.1 ± 0.9 distinct female clones per colony sample. The most dominant haplotypes within a colony were on average found in 3.6 ± 1.1 out of the five sampled female shoots.

A total of 159 different dwarf male haplotypes were found (from 209 samples). No haplotype was found in more than one population. Most dwarf male haplotypes were found only once (80 %), 14 % were found twice and 6 % were found between three and seven times. Five haplotypes occurred in more than one colony but never in more than three colonies within a population. For dwarf males on a single host shoot (mean sample size 10 ± 2.1), the proportion of distinct haplotypes ranged between 40 % (four different dwarf male haplotypes on a single female) and 100 % (12 different dwarf male haplotypes on a single female) with a mean of 80 ± 17 %. Twelve out of 21 colonies in the study contained one or more dwarf males with identical haplotypes as one or more female shoots within the colony. A summary of the sample sizes and number of distinct haplotypes found in each population (as well as colony means) can be found in Table 3.

**Table 3:** Sample sizes and number of SNP-haplotypes in four populations of the moss *Homalothecium lutescens* in Sweden

	Sample size (colony mean ± SD)	
Pop	Female shoots	Dwarf males
AP	25 (5 ± 0)	47 (9.4 ± 2.4)
KP	25 (5 ± 0)	50 (10.0 ± 2.9)
LQ	25 (5 ± 0)	53 (10.6 ± 1.7)
CK	30 (5 ± 0)	59 (9.8 ± 1.6)
Total	105	209
Number of haplotypes (colony mean ± SD)		
Pop	Female shoots	Dwarf males
AP	5 (2.2 ± 0.8)	40 (8.0 ± 2.5)
KP	7 (1.6 ± 0.5)	35 (7.0 ± 1.8)
LQ	3 (2.0 ± 0.0)	40 (8.4 ± 0.8)
CK	9 (2.7 ± 1.4)	44 (8.0 ± 1.1)
Total	22	159

Overall, the population diversity levels were comparable between dwarf males and females within each population. In population AP and LQ, the dwarf males had slightly higher levels of genetic diversity (as measured by uh and %P) than the female shoots (Table 4). Population KP and CK showed the opposite pattern (although the difference was small in population KP) (Table 4).

**Table 4:** Genetic diversity in four populations of the moss *Homalothecium lutescens*

Pop	Category	uh	% *P*
AP	Dwarf males	0.333	66.19
	Female shoots	0.264	54.04
KP	Dwarf males	0.280	56.87
	Female shoots	0.284	57.67
LQ	Dwarf males	0.170	36.81
	Female shoots	0.091	20.33
CK	Dwarf males	0.221	50.52
	Female shoots	0.259	56.47

Values are calculated for dwarf males and female shoots separately. Genetic diversity estimates (uh and %P) are based on 1000 permutations of one random individual per colony sample (*N* = 5 except for in CK where *N* = 6). uh = unbiased diversity (uh = (n/(n-1)) × (1- Σρ^2^)), %*P* = proportion polymorphic loci

At colony level, the mean genetic distance (i.e. proportion of differing alleles) between two distinct female clones (mean over colonies = 34 ± 7 %, *N* = 17 as colonies where all sampled females were identical were not included) were at least as high as the mean genetic distance between a female shoot from the colony and a different female clone from another colony (mean over colonies = 32 ± 6 %) (*F*_[1,16]_ = 9.71, *p* = 0.007). This result means that two distinct female clones sampled from the same colony are at least as different from each other as two distinct clones sampled from two different colonies in a population.

Dwarf male diversity was not significantly associated with female shoot diversity on colony level, either in %P, uh or Hap (%P: *F*_[1,19]_ = 1.528, *p* = 0.232; uh: *F*_[1,19]_ = 2.751, *p* = 0.114; Hap: *F*_[1]_ = 0.981, *p* = 0.335).

## Discussion

We show that dwarf males most likely originate from spores produced on their host shoot, or as a second alternative, spores produced on female shoots in the near vicinity. Although this result suggests that spores are mainly dispersed locally, it appears as though a fraction of the dwarf males break the general pattern. In a few occassions for each population a dwarf male showed a closer ancestry to a different female haplotype than the haplotype of its actual host, sometimes even more strongly related to a female haplotype present in another population, indicating sporadic events of spore dispersal between colonies or populations resulting in dwarf male establishment. The levels of genetic diversity within the dwarf males were relatively high and comparable to that of the female shoots.

### Similar levels of genetic diversity in females and dwarf males

The overall levels of genetic diversity on population level were comparable between dwarf males and female shoots; neither showed a consistent tendency of being higher than the other. Different processes affect the diversity of female shoots and dwarf males. Hence, whether dwarf male diversity is higher or lower than female diversity may reflect local conditions in the four populations, for example weather conditions or disturbance levels (which may affect for example spore dispersal and spore germination).

Previous studies of *H. lutescens* [45] and another perennial pleurocarpous moss, *Hylocomium splendens* [46], have shown that females are likely to follow a repeated recruitment model [47, 48]. Consequently, present female diversity is a result of the conditions for establishment on the ground during the last few years, perhaps even decades or centuries. On average, each colony sample of five shoots from 0.5 m^2^ consisted of two different female clones. This estimate is comparable to previous findings in the perennial pleurocarp *H. splendens* where an average of 2.2 and 2.6 clones per patch (1 dm^2^) has been detected [49, 50]. Other studies on moss clonal diversity in comparable patch sizes have found both lower [51] and higher estimates [52]. The spore production was abundant in all sampled colonies and as indicated by the diversity of the dwarf males, the diversity of the spores appear to be relatively high. Hence, the limited number of female clones within colonies suggests restricted recruitment of spores on the ground. Furthermore, the high differentiation between the female clones within colonies, suggests that females growing side-by-side originate from spores separated by several generations, supporting the hypothesis that recruitment of females is relatively rare. Restricted establishment of spores in mature moss colonies has been shown or suggested in several studies [50, 53–55] and may be a result of chemicals exuded from the large shoots [53, 56, 57], lack of gaps in the dense colonies or herbivory in the protonema phase [58]. The probability of spore establishment likely differs between habitat types (with for example different degrees of disturbance). A study of *H. lutescens* in grazed grasslands showed that shoots sampled right next to each other could locally have a high (48 %) probability of having different isozyme haplotypes [16]. However, in that specific case, clonal mixing appeared primarily to have been caused by local vegetative dispersal.

On the other hand, the dwarf males are short-lived and likely belong to one, or possibly two, generations. Hence, the diversity of the dwarf males is to a great extent dependent on the present genetic diversity of the sexually reproducing females and males as well as the local levels of spore production and dispersal. The lack of a relationship between female and dwarf male genetic diversity at colony level suggests that the diversity of the dwarf males on a host shoot is strongly influenced by the level of sporadic recruitment from outside of the colony. Alternatively, the female clones within a population may contribute unequally to spore production, which would also weaken a potential relationship between female and dwarf male diversity.

Furthermore, the dwarf males may be seen as a form of metapopulation system [59]. The conditions for establishment and survival of dwarf males (for example moisture levels) [4], may fluctuate locally, resulting in different levels of dwarf male abundance or even local extinctions. As long as there is a continuous production of spores in one or more patches within the population, dwarf males may be re-recruited when conditions improve. It is also possible to imagine source-sink dynamics [60] where a few high quality patches may maintain high, stable levels of spore production. Such patches may supply spores to satellite patches where microclimatic conditions do not allow a sustainable dwarf male population. The nature of the dynamics between different patches may have significant consequences for both overall genetic drift and selection [61]. In addition, the success of local vs. recruited male spores may differ among years dependent on the weather and physiological differences among the genomes. To elucidate the dynamics of dwarf male abundances within populations and how it influences for example the effective population size, data on genetic variation in combination with fluctuations of dwarf males on colony-level is needed.

### Dwarf males are most likely produced by their host shoot

Dwarf males were not randomly distributed within populations; they were generally most related to their host shoot, secondly to their colony and then to the rest of the population. In addition, a negative association between kinship and geographic distance was detected for individual dwarf males in three out of four populations. The overall pattern of significant spatial structure in the dwarf males in relation to the females suggests restricted spore dispersal within populations.

This is the first study of genetic structure of moss dwarf males, and as a matter of fact, few comparable studies exist regarding genetic population structure of other groups in which nannandry occurs. A study of *Osedax rubiplumus*, a sessile marine bone worm with epiphytic dwarf males that are dispersed by pelagic larvae, revealed high diversity of males on a single female trunk [13]. In contrast to our results, there was no indication of dwarf males being more related to their female host. Instead, isozyme data suggested that dwarf males were recruited from a vast common larval pool produced by a great number of females. One possible explanation could be that the larval dispersal by ocean currents is more efficient than the spore dispersal by wind in mosses. Alternatively, the *Osedax* females may be able to prevent establishment of closely related males.

Close relatedness between dwarf males and female host shoots has previously been suggested by several authors [11, 18, 24, 25]. Considering the limited fertilization distance of mosses, a significant small-scale genetic spatial structure inevitably leads to some level of inbreeding if no selection (either pre- or post-fertilization) occurs. Our findings of identical female haplotypes in different geographical locations could be an indication of significant inbreeding (or alternatively, significant vegetative dispersal, either present or historical). The fact that a majority of the colonies in the study contained one or more dwarf males with identical haplotype as one or more female shoots within the colony suggests that females occasionally produce spores that are identical (or near identical) to themselves. Identical parent-offspring haplotypes could be a result of either repeated inbreeding or possibly self-fertilization of rare bisexual individuals. However, the latter appears unlikely, as male sexual organs on large individuals are extremely rare in *H. lutescens* and no records of bisexual individuals exist [4, 16, 62]. In accordance with the inbreeding hypothesis, an analysis of the sporophytes on the female shoots confirms that high levels of homozygosity do occur in the studied populations, but varies strongly between shoots [28].

### Dwarf male dispersal

In theory, the protonema from a single spore could give rise to multiple dwarf males with identical haplotype on a female shoot. On the contrary, we found that most dwarf males on a female host shoot belonged to distinct haplotypes (mean 80 %). In addition, the levels of genetic diversity were comparable to that of the females. Several dwarf males showed a high similarity to non-host female shoots in the population (within or outside of the colony) and occasionally another population, suggesting sporadic spore dispersal between colonies and perhaps even populations. Recent studies showed for *Discelium nudum*, that although the colonization rate from spores was higher closer to the mother plant, the majority of spores during a single dispersal event travel beyond the nearest vicinity of the mother and are likely to be deposited over extensive areas (at least in open landscapes) [63, 64]. In addition, several other studies of the colonization of mosses on man-made habitats have shown that although stochastic and rare, long-range dispersal and establishment on a magnitude of tens of kilometres may occur [65–68].

Both dwarf males and female shoots overlapped genetically to some extent between populations, which may have been caused either by dispersal between populations or by common ancestry. The differentiation estimates between populations (*Φ*_PT_ = 0.286 in the dwarf males and 0.374 in the female shoots) were comparable to previous studies of regional diversity in mosses where *F*_ST_ (or comparable differentiation measures) averaged 0.213 for commonly sexual taxa and 0.314 for mainly clonal taxa (review of 30 terrestrial moss species, 25 of which were unisexual and five bisexual) [69]. However, these studies were mostly based on isozyme, RAPD or microsatellite markers; as *F*_ST_ is dependent on the level of total diversity (which may differ depending on the marker used), differentiation estimates may not be directly comparable.

### General consequences of the dwarf male sexual system on population dynamics

Within colonies, recruitment of dwarf males is certainly more frequent than recruitment of females (but note, that populations do exist where dwarf males are rare for one reason or another). Although spores seem to mainly disperse within colonies, exceptions occur. As a result, significant outcrossing may exist side by side with inbreeding. The potential of spores to disperse long distances and establish as dwarf males in high numbers is likely to increase the gene flow between and within nannandrous moss populations. Although females may only sporadically be recruited from spores, the potential of the dwarf males to accumulate high levels of genetic diversity should contribute to the diversity of the clonal (female) part of the population in the long term.

On the other hand, the fact that dwarf males in most species appear to be annual, or at least short-lived, introduces a weakness into the system. If sexual reproduction fails for one or two years in a population (or alternatively, all dwarf males die or fail to establish), no new dwarf males can be locally recruited. In accordance, it is not unusual to find populations of nannandrous mosses where dwarf males are sparse or lacking completely [4, 20, 16]. Although large males do exist in *H. lutescens*, they are extremely rare. Consequently, in a situation where all dwarf males are wiped out from a population, incoming spores from other populations are close to essential to re-initiate sexual reproduction. It is notable that such a rescue effect would mean that a majority of dwarf males are more closely related to females from the source populations than to the females they are colonizing, creating in one blow a strong burst of outcrossing and presumably an inflow of new alleles into the population.

## Conclusions

In this study we show that dwarf males are not randomly distributed within populations. Significant small-scale spatial structure exists where dwarf males are most likely to originate from their host shoot (or at least their own colony). However, a small fraction of the spores that establish on a female shoot appear to have non-host origin. Thus, the dwarf male sexual system appears to simultaneously increase the probability for inbreeding and promote outcrossing. Due to the fact that dwarf males may readily establish in high numbers on a small area, a small fraction of migrants is enough to ensure gene flow between colonies or even populations. In conclusion, the dwarf male sexual system appears to entail an evolutionary advantage by its potential to increase recombination and redistribute the gene pool.

To further elucidate the relative contribution of dwarf males to the genetic structure and diversity, it would be interesting to compare populations with and without dwarf males, for which mosses are an excellent study subject. Furthermore, the unique combination of two fundamentally different recruitment models in females (repeated recruitment) and dwarf males (metapopulation) within the same species, allows for a number of interesting studies on the effects of genetic drift and selection.

## Availability of supporting data

The SNP genotypes of all the samples as well as the sequences for the 68 used SNP markers are available from the Dryad Digital Repository at http://dx.doi.org/10.5061/dryad.1860s. All isotigs used for the SNP extraction are stored in an annotated database available at http://mbio-serv2.mbioekol.lu.se/Mosses.
